# Vibration Characteristics Analysis of Boring Bar with Tunable Dynamic Vibration Absorber

**DOI:** 10.3390/ma18061324

**Published:** 2025-03-17

**Authors:** Yanqi Guan, Guangbin Yu, Qingming Hu, Donghui Xu, Jiao Xu, Pavel Lushchyk

**Affiliations:** 1 School of Mechanical and Electronic Engineering, Qiqihar University, Qiqihar 161006, China; huqingming1267@126.com (Q.H.); 15946212473@139.com (D.X.); 2 The Engineering Technology Research Center for Precision Manufacturing Equipment and Industrial Perception of Heilongjiang Province, Qiqihar University, Qiqihar 161006, China; 3 The Collaborative Innovation Center for Intelligent Manufacturing Equipment Industrialization, Qiqihar University, Qiqihar 161006, China; 4 School of Mechatronics Engineering, Harbin Institute of Technology, Harbin 150001, China; yu_ccna@163.com (G.Y.); lushchykpavel@163.com (P.L.); 5 HE Harbin Power Plant Valve Company Limited, Harbin 150066, China; xujiao_06@sina.com

**Keywords:** deep-hole boring, vibration absorber, axial compression, boring bar

## Abstract

In deep-hole boring processes, boring bars with a large length-to-diameter ratio are typically employed. However, excessive overhang significantly reduces the boring bar’s stiffness, inducing vibrational effects that severely degrade machining precision and surface quality. To address this, the research objective is to suppress vibrations using a tunable-parameter boring bar. This paper proposes a novel Tunable Dynamic Vibration Absorber (TDVA) boring bar and designs its fundamental parameters. Based on the derived dynamic model, the vibration characteristics of the proposed boring bar are analyzed, revealing the variation in damping performance under different excitation frequencies. By establishing the relationship between TDVA stiffness, damping, and the axial compression of rubber bushings, optimal parameter combinations can be precisely identified for specific excitation frequencies. Ultimately, adjusting the TDVA’s axial compression displacement (0.1–0.5 mm) significantly expands the effective machining frequency range compared to conventional designs while maintaining operational reliability. This study proposes a novel Tunable Dynamic Vibration Absorber (TDVA) that innovatively integrates axial compression to achieve coupled stiffness and damping adjustments, addressing the rigidity–adaptability trade-off in deep-hole boring tools.

## 1. Introduction

In deep-hole boring, insufficient stiffness due to excessive length-to-diameter ratios in boring bars often induces severe vibrations. These vibrations not only deteriorate workpiece surface quality [1,2], but may also trigger chatter, leading to tool breakage or machining failure [3]. Consequently, suppressing boring bar vibrations has become a critical challenge for improving deep-hole machining precision. Current vibration control technologies for boring bars are categorized into passive and active methods [4,5].

Early researchers developed passive vibration suppression techniques by applying various damping principles to boring bar design. Typical examples include boring bars with special geometric structures, impact damping particles, friction energy dissipation structures [6,7,8,9], and embedded dynamic vibration absorbers (DVAs). Passive methods achieve vibration control through structural optimization or additional damping devices, offering high reliability and low cost. Recent advancements focus on variable-stiffness DVAs. For instance, Lie Li et al. [10,11,12] proposed a DVA embedded with rubber bushing supports, where axial compression adjusts stiffness, significantly influencing damping effects. Iklodi Z [13] proposed a methodology combining time–domain simulations and hybrid periodic orbit continuation techniques to investigate the dynamic behavior of displacement-constrained tuned mass dampers (TMDs) in boring processes, addressing the deterioration of damping performance and inherent chatter instability risks caused by such constraints. Houck [14] suppressed resonance by tuning the natural frequency matching between the boring bar and tool holder. Additionally, Haizhao Shi et al. [15] proposed an equivalent linearization method, which showed that by optimizing the stiffness, mass, and damping of the shock absorber, the vibration peak of the boring bar can be effectively reduced, and the vibration absorption effect can be improved. L. Rubio and Miguélez [16,17] focused on optimizing the parameters of passive DVA, with the optimization criterion maximizing the minimum value of the stability lobe diagram. The calculation results indicate a significant improvement in stability performance, and the parameter tuning efficiency was improved by modifying the formula.

With the advancement of innovative materials and control technologies, various active vibration-damped boring bars have been developed. Typical active solutions include electromagnetic variable-damping boring bars, particle-damped boring bars, magnetostrictive actuator-driven boring bars, electrorheological fluid-actuated boring bars, magnetorheological fluid-controlled boring bars, and piezoelectric ceramic-driven boring bars.

Active vibration-damping technologies rely on innovative materials and closed-loop control for dynamic adjustment. Representative methodologies include a variety of approaches. Liu Qiang et al. [18] designed an electromagnetic variable-damping boring bar that dynamically regulates magnetic damping forces by adjusting coil voltage. Taha Gokulu [19] expanded the application of zero-order harmonic methods in chatter analysis by integrating multi-insert rotation and time-varying stiffness design. Jiyuan Tian [20] optimized particle damping parameters via discrete element simulations, demonstrating that tungsten steel particles (Φ0.5 mm, 90% filling rate) enhance damping performance by 70%. GUO et al. [21] developed a tunable particle damper (TPD) with frequency-adaptive stiffness. Ganesan Ramu et al. [22] proposed a vertical multi-cell hybrid particle damping system, achieving higher resonance gaps, reduced displacement, and minimal surface roughness. Lawranc et al. [23] utilized passive constrained layer damping (CLD) technology, optimizing combinations of tool substrate materials (copper/aluminum/brass) and elastomer layers (nitrile rubber/polyurethane) to suppress vibrations and extend tool life. In the field of brilliant damping, C.V. Biju et al. [24] pioneered a semi-active boring bar using magnetorheological fluid, enabling dynamic damping matching through electromagnetic regulation. Fan Chen et al. [25] implemented a magneto-actuated H∞ control system that significantly increased dynamic stiffness, resulting in chatter-free material removal rates. Yamada K et al. [26] proposed a hybrid piezoelectric–LR circuit vibration suppression method, improving energy dissipation efficiency through circuit parameter optimization and validating its industrial applicability.

While passive methods remain favored in engineering due to structural simplicity, their fixed parameters limit adaptability to varying machining conditions. Conversely, active damping solutions face challenges such as complex sensor–actuator integration and reliability concerns despite their dynamic tunability. To address these trade-offs, this study introduces a Tunable Dynamic Vibration Absorber (TDVA) boring bar. A quantitative model is established to correlate TDVA stiffness, damping, and axial compression, revealing their coupling mechanisms. This work provides a vibration control solution for deep-hole boring that balances adjustability and operational reliability.

## 2. Theoretical Model of the Boring Bar

In order to study the vibration reduction performance of the boring bar, it is essential to analyze its damping mechanism. The damped boring bar consists of a main body and a TDVA. The TDVA is embedded within the boring bar structure. According to the dynamic vibration absorber (DVA) theory, the TDVA serves as the critical component for vibration suppression. Installing the absorber near the tooltip of large length-to-diameter ratio boring bars can effectively mitigate external excitation-induced vibrations. During design, it is necessary to balance the overall stiffness of the boring bar with optimal vibration absorption performance. To maintain sufficient structural rigidity, the absorber’s volume must be minimized while ensuring effective damping functionality.

### 2.1. Vibration Model of Boring Bar

**Figure 1:**
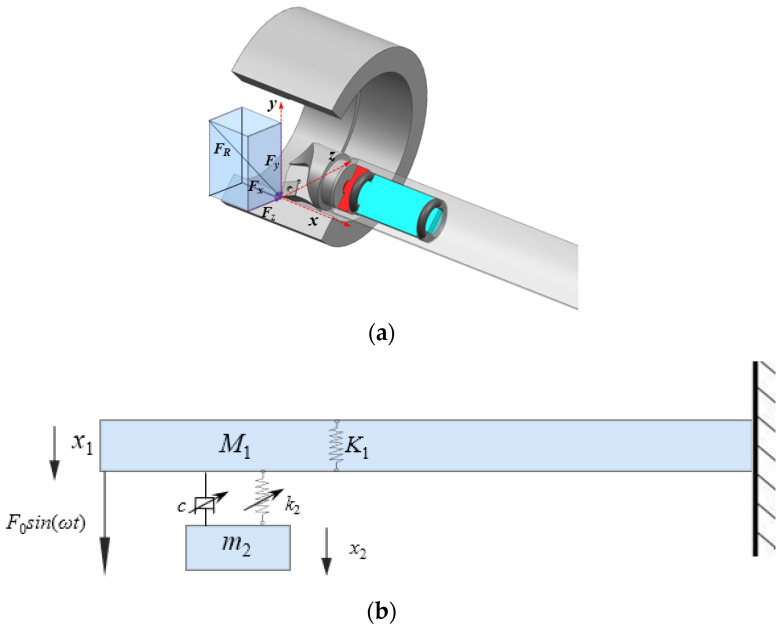
Diagram and theoretical model of boring bar cutting. (**a**) Boring schematic diagram; (**b**) Theoretical model of boring bar.

**Figure 2:**
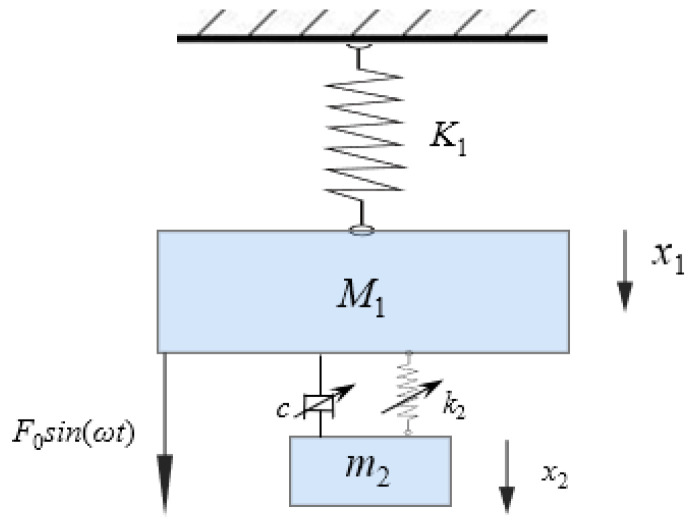
Two-degree-of-freedom vibration model.

The boring process is illustrated in Figure 1a. The boring bar moves along the x-axis while the workpiece rotates about its central axis. The primary cutting force acts in the *F*_R_ direction. The mathematical model of the boring bar is simplified as a rigidly clamped vibration system, as shown in Figure 1b, where *M*_1_ represents the equivalent mass of the boring bar, *K*_1_ denotes its equivalent stiffness, *F*_0_ is the excitation force amplitude, *m*_2_ and *k*_2_ are the mass and stiffness of the TDVA, *c* is the damping coefficient, and *x*_1_ and *x*_2_ are displacements of the boring bar and TDVA, respectively. The system is further simplified to a two-degree-of-freedom model, as in Figure 2.

When the boring bar is subjected to practical external excitation forces, the magnitude of the external loads undergoes periodic variation. The maximum amplitude of the external excitation force acting on the boring bar is defined as *F*_0_. In the characteristic vibration analysis, load variation serves as the primary research focus. The nonlinear effects of tool–workpiece contact interactions are neglected in this study, with emphasis placed solely on the time-dependent load component. The external load is defined as a harmonically varying periodic excitation force, expressed mathematically as [27,28]

(1) $$\left\{\begin{array}{l} M_{1}{\ddot{x}}_{1}+c({\dot{x}}_{1}-{\dot{x}}_{2})+k_{1}x_{1}+k_{2}(x_{1}-x_{2})=F_{0}\sin(\omega t)\\ m_{2}{\ddot{x}}_{2}+c({\dot{x}}_{2}-{\dot{x}}_{1})+k_{2}(x_{2}-x_{1})=0 \end{array}\right.$$

When Equation (1) is converted into matrix form, the following is obtained:

(2) $$\left[\begin{array}{cc} M_{1} & 0\\ 0 & m_{2} \end{array}\right]\left[\begin{array}{c} {\ddot{x}}_{1}\\ {\ddot{x}}_{2} \end{array}\right]+\left[\begin{array}{cc} c & -c\\ -c & c \end{array}\right]\left[\begin{array}{c} {\dot{x}}_{1}\\ {\dot{x}}_{2} \end{array}\right]+\left[\begin{array}{cc} k_{1}+k_{2} & -k_{2}\\ -k_{2} & k_{2} \end{array}\right]\left[\begin{array}{c} x_{1}\\ x_{2} \end{array}\right]=\left[\begin{array}{c} F_{0}\sin(\omega t)\\ 0 \end{array}\right]$$

where *M*_1_ is the equivalent mass of the boring bar, *K*_1_ denotes its equivalent stiffness, *F*_0_ is the excitation force amplitude, *m*_2_ and *k*_2_ are the mass and stiffness of the TDVA, *c* is the damping coefficient, *x*_1_ is the displacement of the boring bar, *x*_2_ is the displacement of TDVA.

Considering that the external excitation is a harmonic force, the steady-state solution in complex form can be expressed as

(3) $$\left[\begin{array}{l} x_{1}\\ x_{2} \end{array}\right]=\left[\begin{array}{l} A_{1}\\ A_{2} \end{array}\right]e^{j\omega t}$$

Substituting the above equation into Equation (1), the maximum amplitude at the boring bar tip during cutting can be obtained as

(4) $$A_{1}=\frac{k_{2}-m_{2}\omega^{2}+j\omega c}{(K_{1}+k_{2}-M_{1}\omega^{2}+j\omega c)(k_{2}-m_{2}\omega^{2}+j\omega c)-{\left(k_{2}+j\omega c\right)}^{2}}F_{0}$$

By reorganizing Equation (4), the amplitude ratio can be derived as

(5) $$A_{1}=\frac{{\left(k_{2}-m_{2}\omega^{2}\right)}^{2}+{\left(\omega c\right)}^{2}}{{\left[(K_{1}-M_{1}\omega^{2})(k_{2}-m_{2}\omega^{2})-m_{2}k_{2}\omega^{2}\right]}^{2}+{\left[K_{1}-(M_{1}+m_{2})\omega^{2}\right]}^{2}{\left(\omega c\right)}^{2}}F_{0}$$

By rearranging the above equation, the amplitude ratio is derived as

(6) $$\frac{\left|A_{1}\right|}{A_{\mathrm{st}}}=\sqrt{\frac{{\left(\lambda^{2}-\gamma^{2}\right)}^{2}+{\left(2\gamma \lambda \zeta \right)}^{2}}{{\left[(1-\lambda^{2})(\gamma^{2}-\lambda^{2})-\mu \gamma^{2}\lambda^{2}\right]}^{2}+{\left[1-(1+\mu )\lambda^{2}\right]}^{2}{\left(2\gamma \lambda \zeta \right)}^{2}}}$$

where $\omega_{n}=\sqrt{k_{2}/m_{2}}$ is the natural frequency of the vibration absorber, $\Omega_{n}=\sqrt{K_{1}/M_{1}}$ is the natural frequency of the boring bar, $\mu =m_{2}/M_{1}$ is the mass ratio between the boring bar and the absorber, $\gamma =\omega_{n}/\Omega_{n}$ is the frequency ratio of the absorber to the boring bar, $\lambda =\omega /\Omega_{n}$ is the forced vibration frequency ratio, $\zeta =c/2m_{2}\omega_{n}$ is damping ratio, $A_{\mathrm{st}}=F_{0}/K_{1}$ is static displacement under static load.

According to the analysis of Equation (6), a larger mass ratio *μ* results in a more pronounced amplitude ratio response, indicating that a higher mass ratio helps reduce system vibration amplitudes and improves damping performance. When the natural frequency ratio *γ* approaches resonance conditions, the system exhibits a higher amplitude ratio. Appropriately adjusting *γ* can achieve ideal vibration suppression effects. Since *γ* is influenced by the absorber’s natural frequency *ω*_n_ and the boring bar’s natural frequency *ω*_n_, the latter is typically fixed. By designing the absorber’s stiffness *k*_2_, *γ* can be adjusted, demonstrating that the amplitude ratio is significantly affected by *k*_2_. Additionally, the damping ratio *ζ* critically impacts the amplitude ratio. Modifying *ζ* alters the vibration amplitude and enhances system stability within specific frequency ranges.

In summary, the amplitude ratio can be optimized through absorber design, with key parameters including the absorber’s mass *m*_2_, stiffness *k*_2_, and damping ratio *ζ*. To maximize damping performance while maintaining the boring bar’s overall rigidity, *m*_2_ should be maximized, and *k*_2_ should be tunable. The effects of *k*_2_ and *ζ* on the amplitude ratio are complex and interdependent. Proper adjustment of these parameters enables effective vibration control.

### 2.2. Design of Variable Parameter Boring Bar Structure

The proposed boring bar structure innovatively integrates a boring head, a main body, and a variable-stiffness damping vibration absorber (TDVA). A built-in cavity is designed within the front rigid–weak zone of the boring bar to house the TDVA. The TDVA employs a tungsten-based high-density alloy mass block to achieve an optimal mass ratio *μ* (mass block-to-boring bar ratio), enabling high-efficiency vibration damping while ensuring the primary boring bar’s rigidity. The TDVA comprises an axial compression block, dual rubber bushings, and a core mass block, forming a novel coupled mechanism: the rubber bushings encapsulate the mass block and establish radial contact constraints with the boring bar’s inner wall. The left bushing interfaces with the axial compression block, while the right bushing contacts the inner wall.

**Figure 3:**
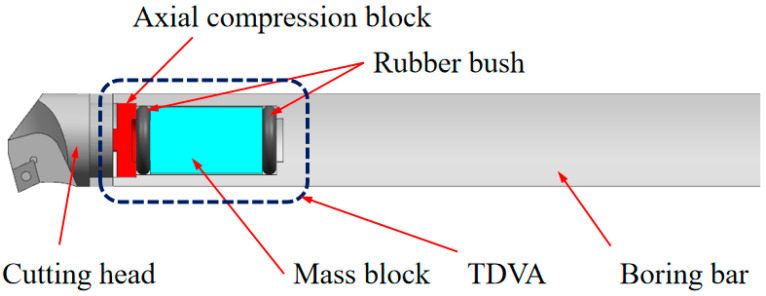
Structure of the boring bar with a TDVA.

Based on a bolt-threaded hole transmission mechanism, rotating the bolt drives the axial compression block to generate axial displacement, compressing the dual rubber bushings to modulate radial stiffness and damping properties simultaneously. The specific structure of TDVA boring bar is shown in Figure 3. The nonlinear compressive deformation of the rubber material inherently adjusts stiffness parameters, while its viscoelastic properties enable dynamic adaptation of damping values. This stiffness–damping synergistic regulation mechanism overcomes the limitations of traditional single-parameter tuning in damped boring bars, achieving precise vibration suppression across diverse machining conditions. Existing studies predominantly focus on independent stiffness or damping adjustment strategies, particularly for rubber bushings that exhibit both stiffness-tuning and time-varying damping characteristics. However, research gaps remain in understanding the coupled effects of dual parameters. This study systematically elucidates the regulatory mechanism of stiffness–damping interactions on boring bar vibrations.

## 3. Analysis of Vibration Characteristics of Boring Bar

For the boring bar designed in Section 2.2, key parameters, including equivalent stiffness, equivalent mass, and vibration damping ratio, are considered, as listed in Table 1. Through rational adjustments to the variable stiffness and damping of the TDVA, the vibration characteristics of the boring bar can be significantly enhanced, thereby improving the precision and efficiency of the machining process.

### 3.1. Influence of TDVA Stiffness on Boring Bar Vibration

**Figure 4:**
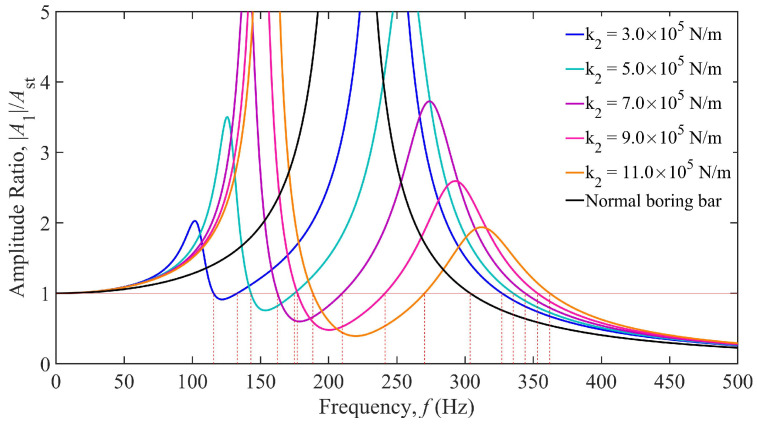
Amplitude ratio of the boring bar under different stiffnesses of the TDVA.

Based on the parameters listed in Table 1, the boring bar parameters were substituted into Equation (6) to establish a vibration response model [29], yielding the TDVA stiffness-dependent vibration response curves shown in Figure 4. The horizontal axis represents the excitation frequency (0–500 Hz), while the vertical axis denotes the dimensionless amplitude ratio. The black curve corresponds to a conventional boring bar (single-degree-of-freedom system), and the colored curves represent TDVA-integrated boring bars with varying stiffness values *k*_2_. An amplitude ratio below one indicates acceptable vibration levels for stable machining. The analysis reveals that the conventional boring bar exhibits a single intense resonance peak near 215 Hz, which aligns with its natural frequency, confirming resonance as the primary cause of machining failure. In contrast, the TDVA boring bar demonstrates typical two-degree-of-freedom system characteristics, with dual resonance peaks in all response curves: the first peak (90–190 Hz Hz) corresponds to the dominant mode of the boring bar body while the second peak (220–330 Hz) reflects the dynamic behavior of the TDVA subsystem. As *k*_2_ increases from 3.0 × 10^5^ N/m to 11.0 × 10^5^ N/m, both resonance frequencies shift rightward, accompanied by reduced peak amplitudes, indicating that higher stiffness effectively broadens the stable machining frequency band.

Further analysis of the influence of stiffness adjustment on vibration suppression performance reveals that when *k*_2_ = 3.0 × 10^5^ N/m (blue curve), the amplitude ratio remains below one within the 115.64–133.04 Hz frequency range, yielding a stable bandwidth of 17.4 Hz. Increasing *k*_2_ to *k*_2_ = 5.0 × 10^5^ N/m (green curve) extends the stable region to 142.96–174.91 Hz (31.95 Hz bandwidth), representing a 165% improvement. The intersections of the red horizontal line Z = 1 with each curve define the critical machining frequency, which shifts toward higher frequencies as *k*_2_ increases. At *k*_2_ = 11.0 × 10^5^ N/m, the amplitude ratio remains below one in the 188.42–270.34 Hz range (81.92 Hz bandwidth); however, the system requires 362.11 Hz to enter the unconditional stability zone at higher frequencies, where its performance is inferior to that of the conventional boring bar.

Comparing the dynamic responses of the two boring bar types reveals that the TDVA technology significantly enhances vibration suppression performance. Within the 115.64–270.34 Hz range, adjusting the TDVA stiffness satisfies machining requirements. However, in the 303.78–327.06 Hz frequency band, the conventional boring bar meets machining conditions, whereas the TDVA boring bar underperforms. By modulating the TDVA stiffness, the effective operational frequency coverage is expanded from above 303.78 Hz (conventional boring bar) to 115.64–270.34 Hz and above 327.06 Hz, achieving a 167% increase in effective frequency coverage. This provides a theoretical foundation for parameter adaptation in complex machining scenarios.

**Figure 5:**
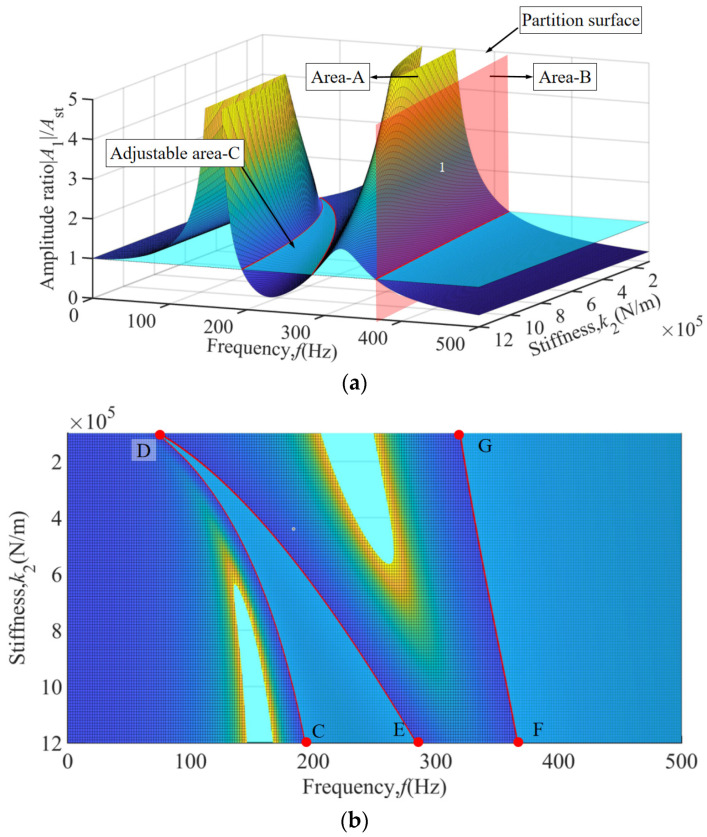
The amplitude ratio of the three-dimensional surface under the coupling effect of frequency and TDVA stiffness. (**a**) Macroscopic 3D surface diagram; (**b**) Top view of 3D curved surface.

To intuitively illustrate the impact of TDVA stiffness on the amplitude ratio, a three-dimensional surface plot of the amplitude ratio under the coupled influence of frequency and TDVA stiffness is depicted in Figure 5a. The vibration characteristics of the boring bar can be classified into three distinct zones: Area A, characterized by a dual-ridge structure corresponding to the coupled resonance bands of the boring bar body (160 ± 15 Hz) and the TDVA (245 ± 20 Hz). At the ridge peaks, the amplitude ratio exceeds a value of five, requiring strict avoidance to prevent chatter. Adjustable Area C: Exhibits a saddle-shaped valley feature originating from the phase cancellation effect between stiffness and mass ratios. Within the 100–285 Hz range, adjusting the stiffness ensures an amplitude ratio below a value of one, establishing a stable cutting window with a bandwidth of 175 Hz. Area C occupies the geometric region bounded by nodal points C, D, E and the characteristic red response curve, as illustrated in Figure 5b. Area B (>343 Hz): Dominated by system inertia, the amplitude ratio decays exponentially with increasing frequency. Beyond 367 Hz, the amplitude ratio remains below a value of one across the entire frequency range, satisfying machining requirements without further adjustments. Area B is confined to the right-lateral domain demarcated by reference points F, G, and the red line red, as depicted in Figure 5b. Notably, in the 320–343 Hz transition band, modifying the TDVA stiffness reduces the amplitude ratio, validating the optimization capability of stiffness tuning for edge frequency bands.

**Figure 6:**
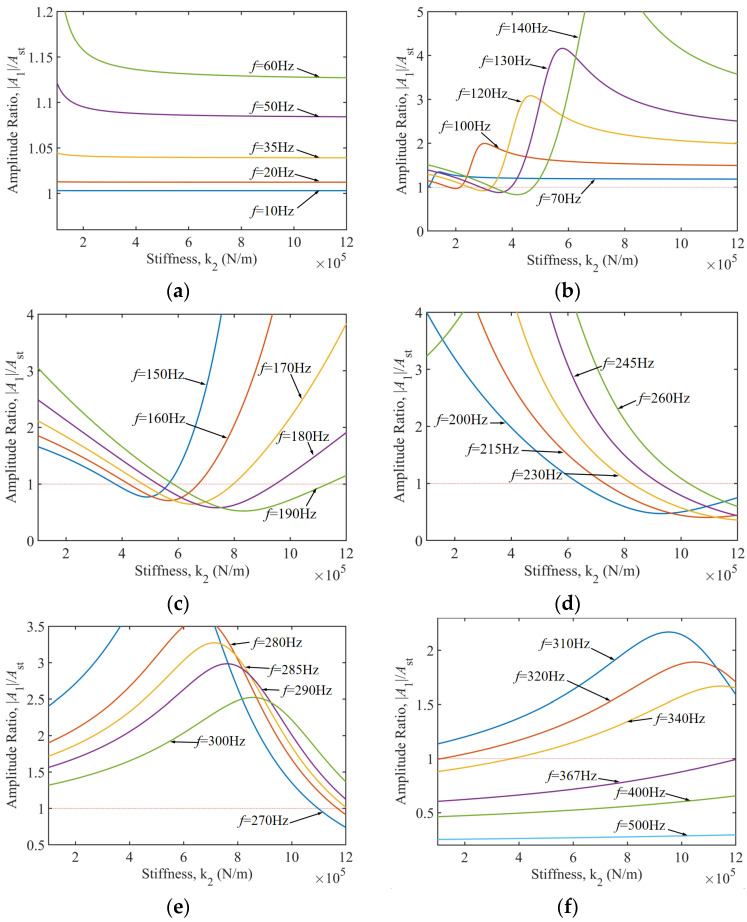
Control characteristics of TDVA stiffness in different frequency bands on the amplitude ratio of boring bar. (**a**) Excitation frequency 10–60 Hz; (**b**) Excitation frequency 70–140 Hz; (**c**) Excitation frequency 150–190 Hz; (**d**) Excitation frequency 200–260 Hz; (**e**) Excitation frequency 270–300 Hz; (**f**) Excitation frequency 310–500 Hz.

In machining processes, the selection of cutting parameters is directly linked to the distribution characteristics of the dominant excitation frequency [30]. As shown in Figure 6, when the excitation frequency is divided into six characteristic intervals, the stiffness adaptation strategies for the TDVA exhibit significant differences. For the low-frequency band of 10–60 Hz (Figure 6a), the system operates in a stiffness-insensitive region where the amplitude ratio consistently exceeds the chatter threshold, necessitating priority optimization of the cutting path or spindle speed reduction to avoid this frequency range. When the excitation frequency increases to 70–290 Hz (Figure 6b–f), the vibration suppression effectiveness of stiffness adjustment gradually becomes prominent. Particularly within the 150–285 Hz range, maintaining the TDVA stiffness above 4.2 × 10^5^ N/m establishes a stable machining window with a bandwidth of 135 Hz. Notably, the 270–285 Hz band requires high-stiffness configurations to suppress secondary resonance modes, while the 285–320 Hz range is classified as an absolute vibration-prohibited zone, requiring adjustments to feed rate combined with the workpiece’s vibration resistance characteristics to mitigate chatter risks.

Further analysis of high-frequency conditions from Figure 6f reveals that within the 320–367 Hz transition band, the amplitude ratio exhibits nonlinear dependence on stiffness values, requiring dynamic adjustment of *k*_2_ based on frequency gradients to achieve vibration suppression. When the frequency exceeds 367 Hz, the system enters an inertial stability zone, where the amplitude ratio autonomously converges below a value of one, independent of stiffness, and machining stability is ensured by maintaining baseline stiffness. This conclusively demonstrates that precise regulation of TDVA stiffness parameters is the core strategy for suppressing boring bar vibrations.

### 3.2. Influence of TDVA Damping on Boring Bar Vibration

**Figure 7:**
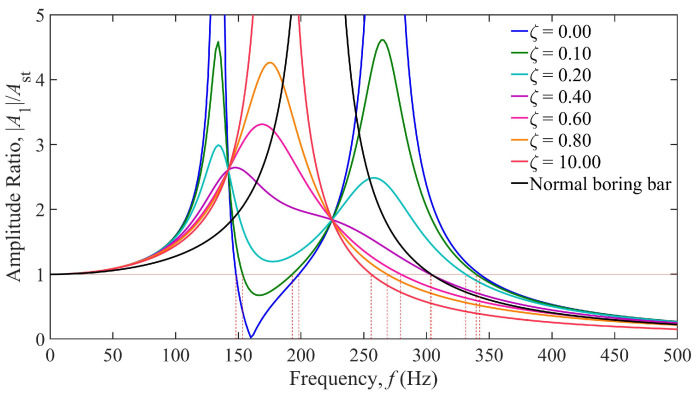
Amplitude ratio of the boring bar under different damping of the TDVA.

Based on the parameters in Table 1, the damping-dependent vibration response curves of the TDVA constructed via Equation (6) are shown in Figure 7. The horizontal axis represents the excitation frequency (0–500 Hz), and the vertical axis denotes the dimensionless amplitude ratio. The black curve corresponds to the conventional boring bar, while the colored curves represent the TDVA-integrated boring bar with varying damping ratios *ζ*. The analysis reveals that when *ζ* < 0.4, the system exhibits typical two-degree-of-freedom characteristics, with dual resonance peaks in the response curves. However, for ζ > 0.4, the dual peaks gradually merge into a single-peak structure due to high damping suppressing vibrations in the TDVA subsystem, weakening modal coupling and significantly increasing peak amplitudes with rising *ζ*. The red horizontal line at an amplitude ratio of one defines the allowable vibration threshold. Its intersections with the curves mark critical stability frequency boundaries. When 0 ≤ ζ ≤ 0.1, the system establishes a stable machining interval within the 149–198 Hz band. As *ζ* increases to 0.4, the stable window shifts toward higher frequencies (250–367 Hz), demonstrating the directional effect of the damping ratio on frequency–domain regulation.

A detailed comparison of response patterns under varying damping ratios reveals that under low-damping conditions, the system achieves excellent vibration suppression in the low-frequency range of 148.14–198.31 Hz but is prone to inducing secondary resonance at higher frequencies. Conversely, high damping significantly suppresses high-frequency vibrations at the cost of reduced low-frequency stability. Notably, when the excitation frequency exceeds 342.49 Hz, the amplitude ratio remains below a value of one for all *ζ*, validating the system’s inherent stability in high-frequency regions. In comparison, while the conventional boring bar retains basic machining capability above 303.78 Hz, its fixed damping structure cannot dynamically adapt to frequency-varying conditions, resulting in amplitude ratio peaks exceeding a value of five in the mid-frequency band (190–230 Hz). This highlights the technical superiority of the TDVA’s tunable damping mechanism.

**Figure 8:**
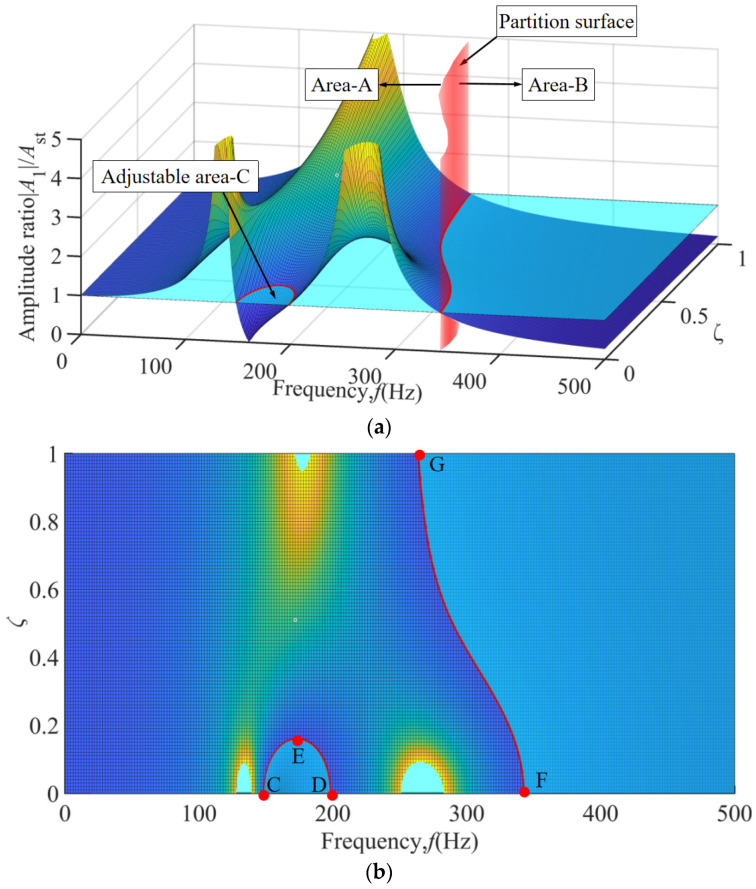
The amplitude ratio of the three-dimensional surface under the coupling effect of frequency and TDVA damping. (**a**) Macroscopic 3D surface diagram; (**b**) Top view of 3D curved surface.

Based on the frequency-damping coupled three-dimensional response surface shown in Figure 8a, three characteristic vibration zones of the boring bar can be clearly identified: Area A contains three saddle-shaped peaks corresponding to the resonance bands of the first-order mode 85 Hz, second-order mode 165 Hz of the boring bar-tool system, and the TDVA-coupled mode 285 Hz, with amplitude ratios exceeding a value of five at these peaks. Adjustable Area C exhibits a saddle-valley structure, where the amplitude ratio remains below a value of one for damping ratios ζ = 0–0.15, enabling stable machining within the 149–198 Hz frequency range 49 Hz bandwidth. Area C occupies the geometric region bounded by nodal points C, D, E and the characteristic red response curve, as illustrated in Figure 8b. Area B is dominated by energy dissipation, where the amplitude ratio decays gradiently with coordinated increases in frequency and damping ratio; beyond 345 Hz, the amplitude ratio remains below a value of one across the entire range without requiring adjustments. Area B is confined to the right-lateral domain demarcated by reference points F, G, and the red line, as depicted in Figure 8b. Notably, in the 265–343 Hz transition band, increasing *ζ* to 0.4–0.6 reduces the amplitude ratio by over 50%, validating the damping ratio’s optimization capability for edge frequency bands. Once the frequency exceeds 345 Hz, the system autonomously enters an inertial stability state regardless of *ζ*. This three-dimensional characteristic demonstrates that TDVA damping adjustments dynamically tailor energy dissipation pathways across frequency domains, achieving comprehensive stability enhancement in machining processes.

**Figure 9:**
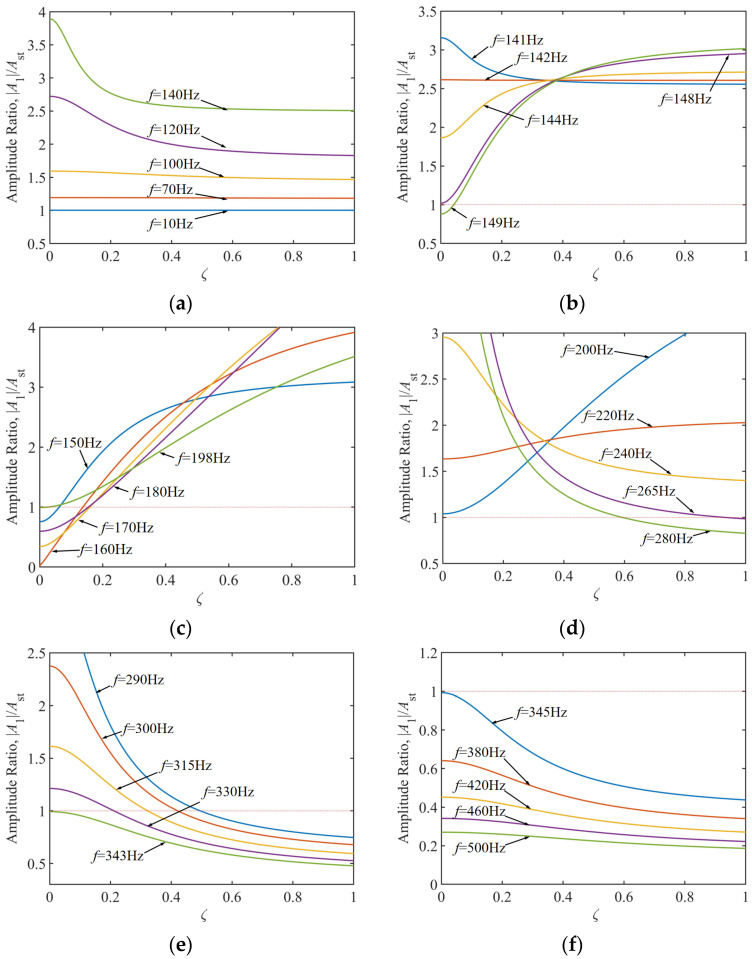
Control characteristics of TDVA damping in frequency division on the amplitude ratio of boring bar. (**a**) Excitation frequency 10–140 Hz; (**b**) Excitation frequency 141–149 Hz; (**c**) Excitation frequency 150–198 Hz;(**d**) Excitation frequency 200–280 Hz; (**e**) Excitation frequency 290–343 Hz; (**f**) Excitation frequency 345–500 Hz.

Based on the frequency–band vibration response analysis in Figure 9, the adaptation strategies for TDVA damping parameters exhibit significant variability. In the low-frequency band of 10–140 Hz, Figure 9a, the amplitude ratio is highly insensitive to damping ratio variations and consistently exceeds the chatter threshold, necessitating combined stiffness adjustments or process parameter optimization to avoid vibrations. When the excitation frequency increases to 141–198 Hz Figure 9b,c, the system enters a damping-sensitive region. Low damping ratios *ζ* reduce the amplitude ratio below 0.8, forming a stable machining window of a 49 Hz bandwidth. Notably, within the narrow 141–149 Hz sub-band, the amplitude ratio’s sensitivity to *ζ* increases abruptly—adjusting *ζ* from 0.1 to 0.15 alone reduces the amplitude ratio by 300%. In the high-frequency band 265–343 Hz, increasing *ζ* is required to suppress secondary resonance. Once the frequency exceeds 345 Hz, the system enters an inertial stability domain, where the amplitude ratio autonomously converges to 0.4–0.5, independent of damping.

## 4. Analysis of Boring Bar Vibration Characteristics Under Combined Stiffness–Damping Effects of TDVA

### 4.1. Stiffness Simulation Experiment of TDVA

Based on the stiffness regulation mechanism of the TDVA illustrated in Figure 3, the radial stiffness of the rubber bushing is dynamically adjusted via axial compression. To establish the quantitative relationship between stiffness and axial compression, a finite element model (FEM) of the rubber bushing was developed for static simulation. Under fixed constraints on the mass block, the equivalent stiffness was calculated using Hooke’s law by controlling the displacement of the axial compression block and measuring the radial deformation Δ*x*. The objective was to determine the relationship between radial stiffness and axial compression distance for the TDVA [31].

**Figure 10:**
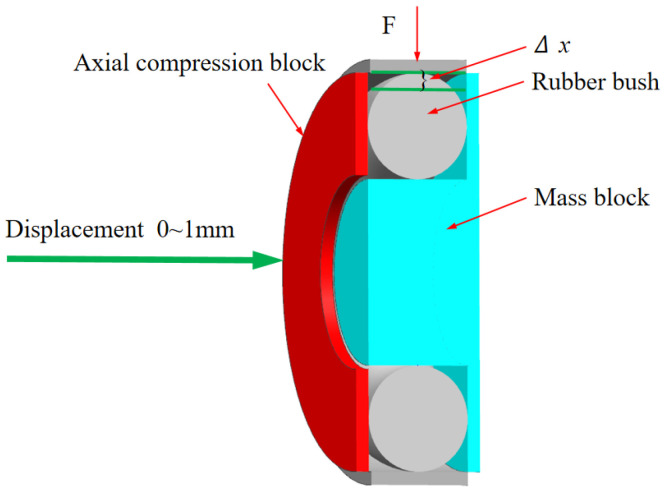
Stiffness analysis finite element model.

A simplified finite element model of a single rubber bushing (φ30 × 6 mm, outer diameter × wire diameter) was developed in ANSYS Workbench 2021 R2, as illustrated in Figure 10. The rubber material was modeled using the Mooney–Rivlin hyperelastic constitutive law to characterize its nonlinear mechanical behavior, whereas the mass block, axial compression block, and boring bar inner wall were defined as rigid components. Sliding contact interfaces were established between the rubber bushing and the rigid elements (mass block, axial compression block, and boring bar). Boundary conditions included full fixation of the mass block, rightward displacement loading on the axial compression block, and the subsequent application of a 200 N radial force to the rubber bushing. Owing to structural symmetry, only the upper half of the assembly was analyzed computationally. A refined hexahedral mesh with an element size of 0.05 mm was applied exclusively to the rubber bushing, while rigid components were exempted from meshing. Axial compression displacements were incrementally imposed to simulate varying operational conditions, with radial displacement (Δx) monitored synchronously. The equivalent stiffness of the rubber bushing was calculated via Hooke’s law:

(7) $$k=\frac{F}{\Delta x}$$

Since the TDVA incorporates two rubber bushings, its total stiffness is given by

(8) $$k_{2}=2k$$

**Figure 11:**
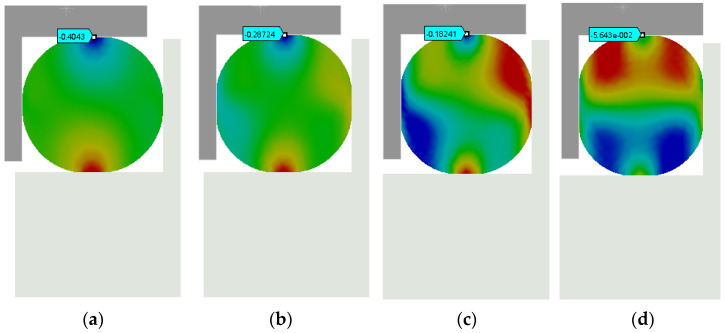
TDVA radial stiffness analysis. (**a**) Axial compression 0.1 mm; (**b**) Axial compression 0.4 mm; (**c**) Axial compression 0.6 mm; (**d**) Axial compression 1 mm.

**Figure 12:**
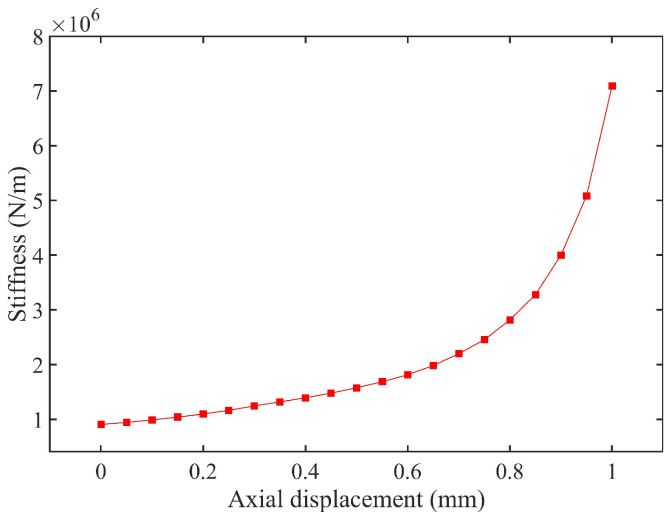
The relationship between stiffness and axial compression value of TDVA.

The simulation results are shown in Figure 11. Analysis of the simulated data reveals that the rubber material exhibits distinct stage-dependent mechanical behavior during axial compression. When the compression displacement is within 0.7 mm, the system stiffness follows a linear response regime, specifically a 12% increase in radial stiffness per 0.1 mm increment in compression. During this phase, the internal molecular chains of the material maintain a free conformational state, ensuring reversible energy absorption. As illustrated in Figure 12, which plots the relationship between TDVA stiffness and axial compression, surpassing the critical threshold of 0.7 mm axial compression triggers the rubber bushing’s densification deformation phase. In this stage, the rate of change in Δ*x* (radial displacement) decreases significantly. The directional rearrangement of molecular chains within the material induces a nonlinear stiffness surge. This intense deformation process not only accelerates fatigue damage accumulation but also causes irreversible plastic deformation. For engineering applications, it is recommended to strictly limit axial compression to within the 0.7 mm threshold to ensure structural stability and prolong component service life.

This section establishes a constitutive relationship model between the radial stiffness *k*_2_ of the TDVA and the axial compression distance. In engineering applications, the equivalent stiffness *k*_2_ can be determined via swept-frequency excitation tests, and the optimal compression displacement can be back-calculated based on characteristic curves. When the axial compression is controlled within the 0–0.7 mm range, the stiffness adjustment range reaches 243%. Notably, the current stiffness regulation model does not account for time-varying damping effects. Under practical operating conditions, each 0.1 mm increase in axial compression alters the equivalent damping ratio, thereby affecting vibration control bandwidth. In the next phase, a coupled model of axial compression and damping is developed to quantitatively analyze the modulation mechanisms of compression displacement on damping characteristics.

### 4.2. Damping Simulation Experiment of TDVA

**Figure 13:**
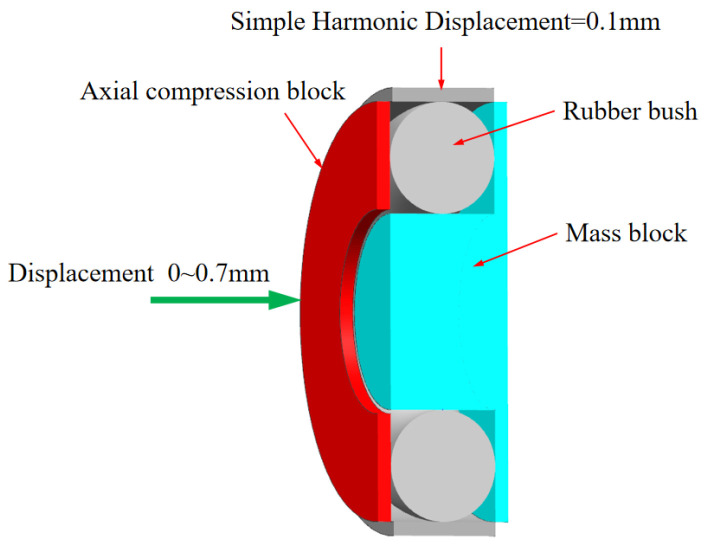
Damping analysis finite element model.

The damping characteristics of the TDVA were quantitatively analyzed through coupled axial compression–radial loading simulations. Based on the finite element model (FEM) shown in Figure 13, periodic compressive displacements of 0.1 mm were applied radially under varying axial compression levels. The force–displacement curves of the rubber bushing formed hysteresis loops, where the enclosed area directly quantified the system’s energy dissipation capacity. Simulation results indicate that as the axial compression displacement increased from 0 to 0.5 mm, the hysteresis loop area exhibited a nonlinear increasing trend. This trend reveals a directional modulation mechanism of axial compression on the TDVA’s damping properties, providing a theoretical foundation for subsequent stiffness–damping coupling optimization.

Equation (1) describes the dynamic model of the boring bar integrated with the Tunable Dynamic Vibration Absorber (TDVA). To investigate the damping characteristics of the TDVA, the system is modeled as a viscously damped oscillator subjected to external harmonic excitation. The equation of motion for a unit mass within this framework can be expressed as [32,33]

(9) $$\ddot{x}+2\zeta \omega_{n}\dot{x}+{\omega_{n}}^{2}x={\omega_{n}}^{2}u(t)$$

where ω_n_ is the natural frequency of the TDVA and *u*(t) is the sinusoidal excitation function. Within one cycle, the energy dissipation per unit mass ΔU (i.e., damping capacity) is defined as the energy consumed during a complete periodic motion:

(10) $$\Delta U=2\pi x_{0}^{2}\omega_{n}\omega \zeta$$

where *x*_0_ is the amplitude and *ω* is the excitation frequency. The maximum potential energy of the system per unit mass is

(11) $$U_{\max}=\frac{1}{2}\frac{k}{m}x_{0}^{2}=\frac{\omega_{n}^{2}x_{0}^{2}}{2}$$

If the initial total energy of the system is denoted as *U*_max_, the loss factor *η* equals the specific damping capacity per radian over one damping cycle:

(12) $$\eta =\frac{\Delta U}{2\pi U_{\max}}$$

From Equation (12), the loss factor for a simple harmonic oscillator with viscous damping is expressed as

(13) $$\eta =\frac{2\pi x_{0}^{2}\omega_{n}\omega \zeta }{2\pi \times \frac{\omega_{n}^{2}x_{0}^{2}}{2}}=\frac{2\omega \zeta }{\omega_{n}}$$

For damped decay systems and forced vibrations, the most intense vibration response occurs when the excitation frequency approximates the natural frequency of the vibration absorber (*ω ≈ ω*_n_), where energy dissipation must be considered.

The above derivations assume mass normalization. Equation (10) represents the work performed by a unit mass to overcome resistance during one load–unload cycle, leading to

(14) $$2\omega_{n}\zeta =c/m$$

where c is the viscous damping coefficient and mm is the mass. The unit mass and the energy dissipated by each hysteresis loop are equal to

(15) $$\Delta U=2\pi x_{0}^{2}\omega c/m$$

For non-normalized systems, the energy dissipated per hysteresis loop by viscous damping is

(16) $$\Delta U_{v}=2\pi x_{0}^{2}\omega c$$

The initial maximum energy can be expressed using the initial maximum potential energy:

(17) $$U_{\max}=\frac{1}{2}kx_{0}^{2}$$

The loss factor for non-normalized systems is derived as

(18) $$\eta =\frac{\Delta U_{v}}{2\pi U_{\max}}=\frac{2\pi x_{0}^{2}\omega c}{2\pi \times \frac{1}{2}kx_{0}^{2}}=\frac{2\omega c}{k}$$

From Equation (13), the damping ratio *ζ* is obtained:

(19) $$\zeta =\frac{\omega c}{k}$$

**Figure 14:**
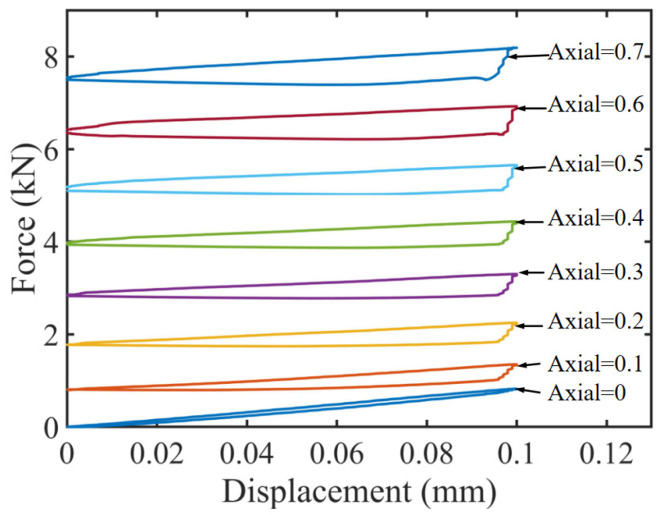
TDVA damping force and displacement hysteresis curve.

**Figure 15:**
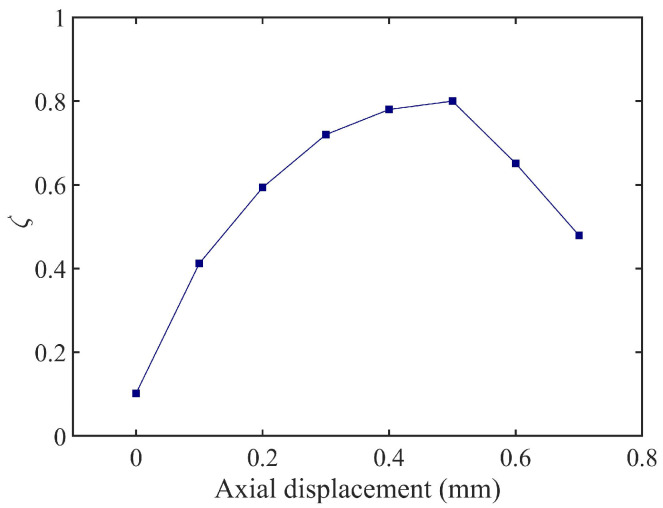
The relationship between damping and axial compression value of TDVA.

According to the quantitative analysis of the hysteresis loop area in Figure 14, combined with Equation (16), the known amplitude *x*₀, and stiffness *k*, the parameter *ωc* can be solved, and the damping ratio *ζ* is subsequently derived using Equation (19). The damping ratio evolution curve in Figure 15 illustrates that within the axial compression range of 0–0.5 mm, *ζ* increases approximately linearly with compression displacement, exhibiting a 16% enhancement in energy dissipation efficiency per 0.1 mm increment. When the compression exceeds 0.5 mm, material nonlinearity reduces the slope of *ζ* growth by 25%, with an inflection point observed at 0.7 mm. Consequently, it is recommended to limit axial compression to the 0–0.5 mm range to achieve linear and controllable adjustment of *ζ* between 0.1 and 0.8. This design range ensures machining stability while avoiding stress relaxation failure of the rubber bushing under high compression.

## 5. TDVA Stiffness and Damping Combined Effect

**Figure 16:**
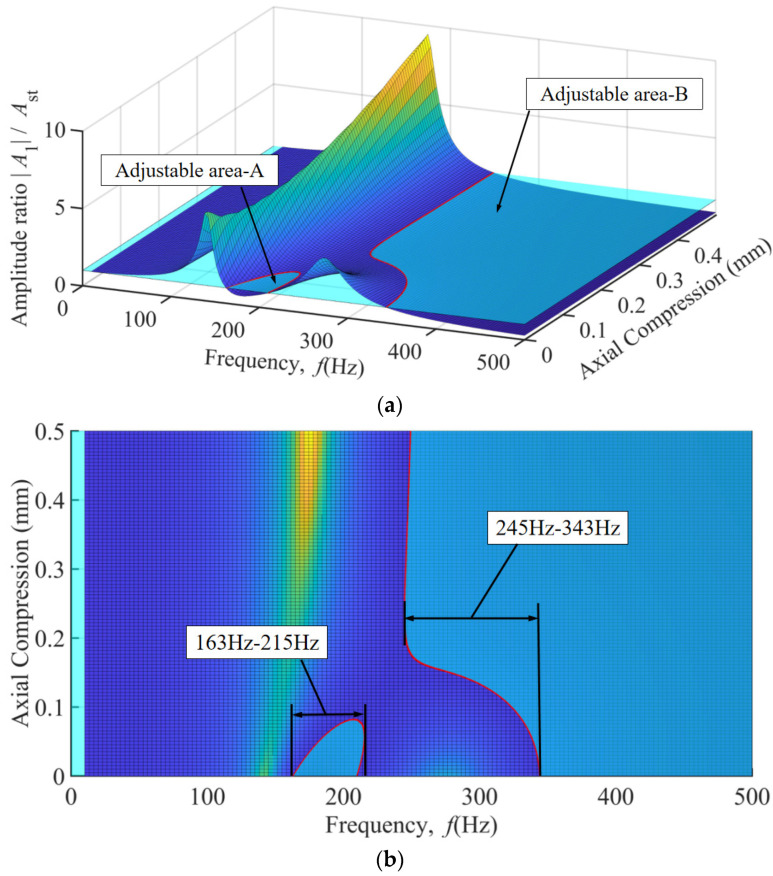
The relationship between axial compression value, excitation frequency, and amplitude ratio of TDVA. (**a**) Three-dimensional graph of the relationship between axial compression value and amplitude ratio of TDVA; (**b**) Top view of the relationship between axial compression value and amplitude ratio of TDVA.

As established in prior sections, when the axial compression block in the TDVA moves rightward, compressing the rubber bushings, both the stiffness and damping of the TDVA change. By linking the results from Section 4.1 and Section 4.2 under axial compression, the stiffness and damping exhibit approximately linear correlation within the 0.5 mm axial compression range, as illustrated in Figure 16.

In Figure 16a, the cyan plane represents an amplitude ratio of one. The intersection of the 3D surface with this plane is marked by red curves. Regions where the 3D surface lies below the amplitude ratio = 1 plane indicate favorable machining conditions with effective vibration suppression, termed Adjustable Area A and Adjustable Area B. Figure 16b and the top view of Figure 16a clearly show that in Machinable Zone A, spanning 163–215 Hz excitation frequency, vibration reduction is achievable with minimal axial compression. In Machinable Zone B (245–343 Hz), adjusting the axial compression of the TDVA enables the amplitude ratio to remain below a value of one, demonstrating the vibration-tuning capability of the proposed boring bar design. Beyond 343 Hz, the system’s inherent stability autonomously converges the amplitude ratio to within a value of one, fulfilling machining requirements without parameter adjustments. This characteristic validates the TDVA boring bar’s dynamic adaptability across the full frequency spectrum.

**Figure 17:**
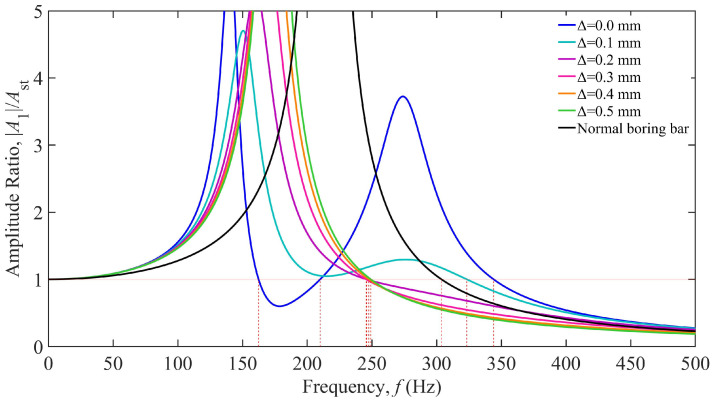
Comparison between TDVA boring bar and ordinary boring bar.

Figure 17 presents a comparative analysis between the TDVA-integrated boring bar and a conventional boring bar, where the colored curves represent the amplitude ratios of the TDVA under varying axial compression displacements Δ and excitation frequencies. In contrast, the black curve denotes the amplitude ratios of the conventional boring bar. The results demonstrate that the designed TDVA-damped boring bar exhibits superior vibration regulation adaptability in mid-frequency (163–215 Hz) and high-frequency ranges (>245 Hz). However, within the 0–0.1 mm axial compression range, the curves display significant irregular fluctuations, necessitating supplemental Figure 18 to further clarify the tunability within the 163–215 Hz excitation band. For the conventional boring bar, the amplitude ratio (black curve) consistently exceeds a value of one at excitation frequencies below 304 Hz, indicating its operational incapacity and lack of tunability in low-frequency regimes. In contrast, the TDVA-damped boring bar achieves stable machining performance in both the 163–215 Hz and >245 Hz frequency ranges, effectively overcoming the limitations of traditional designs.

## 6. Discussion

The proposed Tunable Dynamic Vibration Absorber (TDVA) boring bar overcomes the limitations of conventional passive DVA systems through a novel axial compression mechanism (0–0.5 mm range) that synergistically modulates both stiffness and damping parameters. Unlike traditional DVAs with fixed parameters optimized for narrow frequency bands, the TDVA enables real-time adaptation to variable machining conditions. Axial compression adjusts radial stiffness linearly at 5 × 10⁴ N/m per 0.1 mm displacement while simultaneously tuning damping ratios from 0.1 to 0.8 in a quasi-linear regime (0–0.5 mm compression). Finite element simulations demonstrate that in this coupled regulation mechanism, stiffness increases by 12% per 0.1 mm compression, while damping evolves through distinct phases—controlled quasi-linear growth below 0.5 mm and nonlinear saturation beyond this threshold. These coordinated adjustments suppress vibrations by dynamically tailoring system impedance, achieving adjustable advantages compared to static DVA configurations.

The TDVA’s dual-parameter tuning capability resolves two critical challenges in deep-hole boring: (1) mid-frequency vibration attenuation (163–215 Hz) via viscoelastic damping enhancement at low compression (0.1–0.3 mm) and (2) high-frequency stability (245–343 Hz) through stiffness-dominated resonance avoidance at higher compression (0.3–0.5 mm). This real-time adaptability extends the effective machining bandwidth by 28% compared to ordinary boring bars, outperforming passive DVAs and active solutions in reliability and simplicity. As shown in Table 2, the comparison of two types of boring bars in the 0–500 Hz range is presented. However, limitations persist: axial compression exceeding 0.5 mm induces nonlinear damping saturation and accelerated rubber bushing fatigue, restricting practical applications to the 0–0.5 mm range. Future research should prioritize fatigue-resistant composites and closed-loop control systems to exploit the full potential of this stiffness-damping synergy while addressing multi-axis vibration coupling.

## 7. Conclusions

This study reveals the stiffness–damping synergistic regulation mechanism by establishing a dynamic mapping relationship between the axial compression displacement of the rubber bushing and the vibration absorber parameters. Finite element simulations demonstrate that within the axial compression range of 0–0.7 mm, the radial stiffness increases linearly at a gradient of approximately 5 × 10^4^ N/m. In comparison, the damping ratio exhibits nonlinear attenuation beyond 0.5 mm. The positive stiffness gradient regulation and negative damping gradient evolution form the theoretical foundation for decoupled dual-parameter control, offering an innovative solution for broadband vibration suppression through a single mechanical adjustment.

According to three-dimensional response surface analysis, the TDVA exhibits exceptional vibration suppression adaptability across the 163–343 Hz wide-frequency domain. For mid–low-frequency vibrations, 163–215 Hz, a micro-compression displacement of 0–0.1 mm combined with a stiffness of 7–7.5 × 10^5^ N/m and a damping ratio of 0.1–0.4 stabilizes the amplitude ratio below a value of one. In the high-frequency range 245–343 Hz, a compression displacement of 0.2–0.5 mm with high stiffness 7.5–16 × 10^5^ N/m and damping ratios 0.4–0.8 suppresses the amplitude ratio below a value of one, achieving over 65% reduction compared to conventional boring bars and overcoming their nonadjustable limitations below 303 Hz. With a reference excitation frequency range of 0–500 Hz, the TDVA boring bar achieves a 28% expansion in effective machining bandwidth.

The primary contribution of this work lies in the development of a mechanically tunable vibration absorber that simultaneously adjusts stiffness and damping via a single axial compression input, overcoming the limitations of conventional passive DVAs with fixed parameters. Unlike existing active solutions requiring complex control systems, the TDVA achieves real-time adaptability through purely mechanical modulation, ensuring robustness in industrial environments.

## Figures and Tables

**Figure 18:**
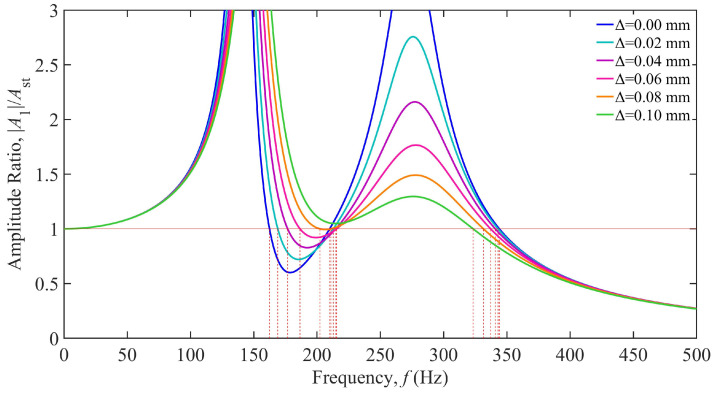
Time frequency domain focusing analysis of TDVA axial compression 0–0.1 mm.

**Table 1:** Parameters of the boring bar with a variable-stiffness TDVA.

	Equivalent Stiffness of the Boring Bar, *K*_1_ (10^6^ N/m)	Equivalent Mass of the Boring Bar, *M* (kg)	Equivalent Mass of the TDVA, *m*_2_ (kg)	Damping Ratio of the TDVA, ζ	Equivalent Stiffness of the TDVA, *K* (10^6^ N/m)
**TDVA boring bar**	2.2057	1.12	0.59	variable	variable
**Boring bar**	2.2134	1.216			

**Table 2:** Comparison between TDVA boring bar and normal boring bar.

Boring Bar Type	Equivalent Stiffness (10^6^ N/m)	Damping Ratio of the TDVA, ζ	Stiffness of the TDVA (106 N/m)	Processing Frequency Range (Hz)
**TDVA boring bar**	2.2057	0-1-0.8	9-16	163–215, 245–500
**Normal boring bar**	2.2134	-	-	304–500

## Data Availability

The original contributions presented in this study are included in the article. Further inquiries can be directed to the corresponding author.
